# Parenchymal neuro-sonological characteristics in epileptic patients and their correlation with cognitive dysfunction

**DOI:** 10.1186/s42494-025-00212-8

**Published:** 2025-04-21

**Authors:** Hanan Amer, Hanan Helmy, Enji El-Sawy, Maha S.Ayoub, Nesma Mounir

**Affiliations:** Department of Neurology, Faculty of Medicine, Cairo University, Cairo, 11562 Egypt

**Keywords:** Epilepsy, Cognition, ACE- III, Third ventricle, Brain atrophy, TCS

## Abstract

**Background:**

Idiopathic generalized epilepsies (IGEs) are the most common syndromes within the “genetic generalized epilepsies” (GGEs). Patients with IGE often exhibit cognitive comorbidities. The primary objective of this study is to investigate the correlation between brain parenchymal sonography characteristics and cognitive impairment in IGE.

**Methods:**

This study enrolled 26 patients with IGE and 26 age- and sex-matched controls. All participants underwent comprehensive evaluations including clinical examination, electroencephalography, magnetic resonance imaging epilepsy protocol, transcranial sonography (TCS) for third and lateral ventricular diameter measurements, and cognitive assessment using the Addenbrooke’s Cognitive Examination-III (ACE III).

**Results:**

This study found significantly lower scores in attention, memory, fluency, and total score of ACE-III in IGE patients compared to the control group (*P*-value = 0.011, 0.033, 0.007, and 0.001, respectively). However, no significant differences were observed between IGE patients and the control group in language and visuospatial score (*P* = 0.479 and 0.108, respectively). The average diameters of the third ventricle and lateral ventricle anterior horns were significantly larger in patients than in the control group (*P*-value 0.004, 0.009, and 0.012, respectively).

**Conclusions:**

IGE patients exhibit significant cognitive impairment and notable dilatation of the third ventricle and lateral ventricles horns, which may serve as markers of brain atrophy.

## Background

Epileptic disorder is a common chronic brain disorder affecting a large population worldwide [1]. The most common syndromes within the genetic generalized epilepsies (GGEs) are idiopathic generalized epilepsies (IGEs) [2]. A broad range of functions within the frontal lobe of the brain are affected by the neuropsychological impairments associated with IGE. Working memory, visuospatial reasoning, response inhibition, verbal fluency, and sustained attention have been found to be impaired in patients with juvenile myoclonic epilepsy (JME) [3]. Transcranial B-mode sonography (TCS) has been used to visualize the brain parenchyma and the intracranially ventricular system through a sonographic approach. It aids in diagnosing different neurosongraphic changes in different neurological diseases [4]. It has been proven to be an accurate, easy and non-invasive imaging modality for this purpose. Also, the width of third ventricle and the lateral ventricles frontal horns were related to cognitive performance, suggesting that their enlargement could serve as a marker for cognitive impairment [5].

Cognitive impairment in IGE has been addressed in previous research [6]. This impairment has significant educational and functional implications for epileptic patients [7]. Previous studies have investigated the sonographic assessment of parenchymal structures in JME and found associations with certain cognitive domains [8, 9]. However, the role of TCS in early detection of cognitive decline in IGE remains under investigation.

This study is a case-control study aimed at detecting cognitive dysfunction in patients with IGE and correlating it with brain parenchymal sonography as an indicator of brain atrophy.

## Methods

This study is a case–control study conducted on 26 IGE patients, diagnosed according to the updated diagnostic criteria for IGEs established by the ILAE’s Nosology and Definitions Taskforce [2] and 26 healthy subjects as a control group. All participants (patients and control) had basic educational skills (ability to read, write, and perform simple calculations), and had completed at least 6 years of education (primary school).This ensured that participants could complete the basic tasks required for the cognitive scale testing. The study aimed to correlate the brain parenchymal sonography to the cognitive assessment in IGE patients.

### Inclusion criteria

1) Epileptic patients with IGE aged 18 to 40 years, of both sexes. 2) Participants with the ability to read, write, and perform simple calculations.

### Exclusion criteria

1) Age below 18 years. 2) Patients with other types of epilepsy (e.g., focal epilepsy, epileptic encephalopathy, or other epilepsy syndromes). 3) Subjects with history of systemic disease that could affect cognition (e.g., renal or hepatic impairment, uncontrolled diabetes). All patients underwent a thorough clinical assessment.

### Electroencephalogram (EEG)

Conventional interictal EEG was performed under standard conditions using the 10–20 international system for at least 30 min, with provocation techniques (photic stimulation and hyperventilation). Recordings were done through Galileo Software (Firenze, Italy) and EB Neuro Basis BE Hardware (Florence, Italy). Brain magnetic resonance imaging (MRI) (1.5 Tesla) was performed for all patients to exclude structural focal lesions and space-occupying lesions.

### Cognitive examination

Cognitive examination was conducted using the Addenbrooke's Cognitive Examination-III (ACE-III) in Egyptian Arabic version. Patients were required to be free of seizures for at least 4 weeks prior to testing. The ACE-III consists of 19 tests assessing five cognitive functions: attention, memory, visuospatial processing, language and fluency. The data of each test is scored to a total score out of 100 (26 for memory, 26 for language,14 fluency, 18 points for attention, and 16 for visuospatial processing). The test takes about 20 min to perform, with higher scores indicating better cognitive performance.

### Transcranial sonography 

**Fig. 1:**
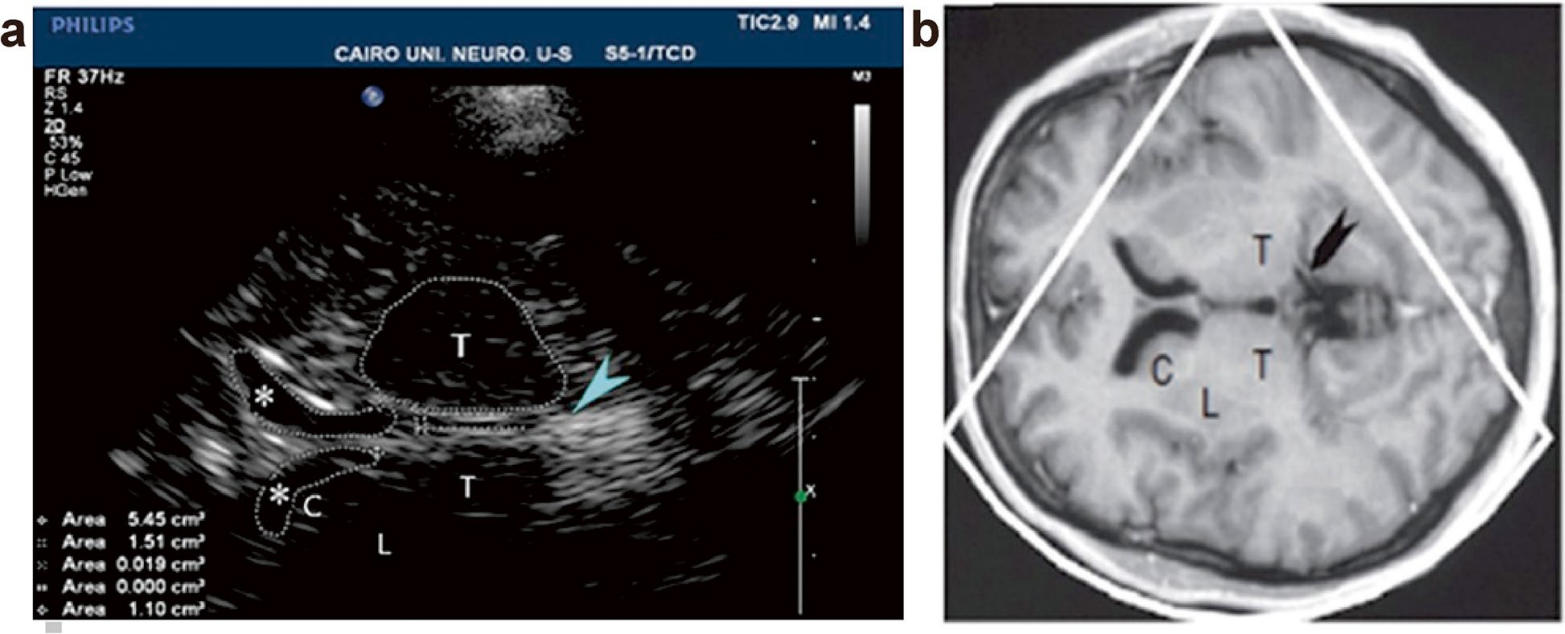
TCS axial view at the thalamus level (**a**) and corresponding MRI picture at the same level (**b**); T = thalamus; * = frontal horn of lateral ventricle; C = caudate nucleus and lentiform nucleus; arrowhead = pineal gland (hyperechogenic landmark)

TCS was performed by an experienced neuro sonographer using the PHILIPS IU22 xMATRIX ultrasonography instrument (L 1–5 transducer California, US), prepared with a 2.5 MHz transducer (phased array). TCS was used to examine the diameter of the third ventricle and bilateral frontal horns as parameters for brain atrophy, compared to matched healthy controls. The sonographic examination was performed in ventricular plane (plane of the thalamus): the start point was the axial midbrain plane, by tilting the transducer about 20°; the pineal gland, identified by its high echogenicity due to calcification, was the landmark structure. The assessed structures included the third ventricle and bilateral frontal horns (Fig. 1) [10]. The third ventricle and each frontal horn appeared as two highly echogenic lines (representing hyperechogenic ependyma) surrounding hypo- or an-echogenic signal of the cerebrospinal fluid (CSF). Each frontal horn was examined contralateral to the insonation side, with the choroid plexus visible as a pulsating structure within each of them [10].

### Statistical methods

Sample size was calculated using Epi-calc 2000, considering 80% power, a 0.05 significance level, a mean of 0.59 in epileptic patients, a mean of 0.38 in normal subjects, and a standard deviation of 0.26. The calculated sample size was 48 (24 per group). Considering the drop-out rate of 10%, the final sample size will be 52 (26 in each group). The Statistical Package of Social Sciences (SPSS) was utilized to examine the results (version 28). The data normality was examined using the Kolmogorov–Smirnov single-sample test. Numerical variables were displayed as mean and standard deviation (SD) and median (range). Qualitative data are described as numbers and percentages. To compare between groups, the Mann–Whitney test was utilized. Spearman correlation was utilized in continuous data correlation. A *P*-value ≤ 0.05 was considered statistically significant.

## Results

This study included 26 IGE patients and 26 age- and sex-matched healthy controls. There was no significant difference in years of education between the patient and control groups (Table 1). Patients were subjected to clinical examination, EEG, MRI epilepsy protocol, third and lateral ventricles diameter measurements using TCS and cognitive assessment using the ACE-III. Regarding seizure types, 46% (12/26) of patients were completely controlled, 38% (10/26) experienced combined myoclonus and generalized tonic–clonic seizure (GTCs), 11.5% (3/26) of experienced generalized tonic–clonic seizures only, and 3.8% (1/26) experienced only myoclonus. The frequency of seizures over the last two years is displayed in Table 1.

**Table 1:** Demographics and clinical data among the studied groups

**Characteristic**	**Patients** **(*n*** **=26)**	**Control** (***n*=26)**	***P*-value**
**Demographics**			
Gender, *n* (%)			0.163
Female	14 (53.8)	9 (34.6)	
Male	12 (46.2)	17 (65.4)	
Age (years), mean ± SD	27.1 ± 6.9	28.5 ± 7.3	0.475
Education, *n* (%)			0.158
6–12 years	18 (69.2)	13 (50.0)	
>12 years	8 (30.8)		
**Clinical data of epileptic patients**			
Epilepsy type, *n* (%)			
Generalized tonic-colonic	15 (57.7%)	–	
Juvenile myoclonic epilepsy	11 (42.3%)	–	
Family history			
Negative	22 (84.6%)	–	
Positive	4 (15.4%)	–	
Epilepsy duration (years), median (range)	7.5 (3–23)	–	
Total Seizure frequency in last 2 years, (mean ±SD)	14.96 ± 25.94	–	
Frequency of GTCs in last 2 years, (mean ±SD)	7.04 ± 11.71	–	
Frequency of myoclonus in last 2 years, (mean ±SD)	6.23 ± 13.65	–	
EEG, n (%)			
Normal	20 (76.9)	–	
Abnormal	6 (23.1)	–	
Fits control, *n* (%)			
Uncontrolled	18 (69.2)	–	
Controlled	8 (30.8)	–	

ACE-III scores in patients and control groups (Table 2) revealed a significant lower score in attention, memory, fluency, and total score of ACE-III in patients compared to the control (*P*-value = 0.011, 0.033, 0.007, and 0.001 respectively). However, no significant differences were observed between patients and controls in language and visuospatial scores (*P*-value = 0.479 and 0.180, respectively). A significantly higher average dilation was observed in the third ventricle, right anterior horn of the lateral ventricle, and left anterior horn of the lateral ventricle in patients compared to controls (*P*-value 0.004, 0.009, and 0.012, respectively) (Table 3). However, no significant correlation was found between the diameter of the third ventricle or bilateral anterior horn of lateral ventricles and attention, memory, fluency, language, visuospatial, or total ACE-III scores.

**Table 2:** Comparison of ACE-III scores between cases and control

	**Total (*n* = 52)**	**Cases (*n* = 26)**	**Control (*n* = 26)**	***P*-value**
**Attention**				
Mean ± SD	16.5 ± 2.1	15.7 ± 2.4	17.2 ± 1.3	
Median (range)	18 (10–18)	17 (10–18)	18 (14–18)	0.011*
**Memory**				
Mean ± SD	19.4 ± 3.5	18.4 ± 3.4	20.4 ± 3.4	
Median (range)	20 (13–25)	18.5 (13–25)	21 (13–25)	0.033*
**Fluency**				
Mean ± SD	7.8 ± 3.1	6.5 ± 3.5	9 ± 1.9	
Median (range)	8 (0–13)	6.5 (0–13)	9 (6–12)	0.007*
**Language**				
Mean ± SD	23.2 ± 2.5	22.7 ± 3.2	23.7 ± 1.5	
Median (range)	24 (13–26)	24 (13–26)	24 (20–26)	0.479
**Visuospatial**				
Mean ± SD	14.04 ± 2.2	13.4 ± 2.6	14.6 ± 1.6	
Median (range)	15 (8–17)	14 (8–17)	15 (11–16)	0.108
**Total**				
Mean ± SD	80.9 ± 9.7	76.8 ± 9.5	84.9 ± 8.1	
Median (range)	82 (53–94)	80 (53–90)	88 (68–94)	0.001*

*SD* standard deviation; *P value ≤ 0.05 is significant; *n* number

**Table 3:** Comparison of parenchymal brain ultrasound between patients and controls

	**Patients (*n* = 26)**	**Control (*n* = 26)**	***P*-value**
**Third Ventricle (cm)**			
Mean ± SD	0.3 ± 0.3	0.1 ± 0.03	
Median (range)	0.15 (0.1–0.9)	0.1 (0.07–0.23)	0.004*
**Right Anterior Horn of the lateral ventricle**			
Mean ± SD	0.27 ± 0.26	0.1 ± 0.04	
Median (range)	0.15 (0.1–0.9)	0.1 (0.08–0.26)	0.009*
**Left Anterior Horn of the lateral ventricle**			
Mean ± SD	0.27 ± 0.25	0.1 ± 0.05	
Median (range)	0.16 (0.1–0.9)	0.12 (0.08–0.29)	0.012

*SD* standard deviation; *P value ≤ 0.05 is significant; *n* number

**Fig. 2:**
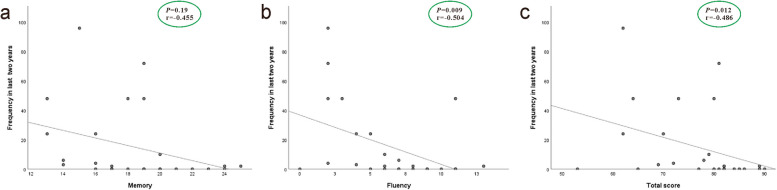
Correlation between frequency of seizures in the last two years and cognitive assessment. **a** Correlation between frequency of seizures in the last two years and Memory. *P* = 0.19, r = −0.455. **b** Correlation between frequency of seizures in the last two years and Fluency. *P* = 0.009, r = −0.504. **c** Correlation between frequency of seizures in the last two years and total ACE score. *P* = 0.012, r = −0.486

A significant correlation was found between memory, fluency, and total ACE-III scores and the frequency of seizures over the last two years (*P*-value = 0.019, 0.009, and 0.012, respectively) (Fig. 2). In contrast, no significant correlation was detected between attention, language or visuospatial scores and seizure frequency. (*P*-value = 0.939, 0.925 and 0.286 respectively). Additionally, no significant correlation was observed between ventricular dilatation and seizure frequency over the last two years in patients’ group.

Regarding the relation between the level of education and ACE-III scores in the patient group, there was no statistically significant difference in ACE-III scores between the group of patients less than 12 years of education and those with more than 12 years of education (Table 4).

**Table 4:** Relation between level of education and ACE-III scores in patients’ groups

ACE-lll scores	Group 1 (6–12) years (*n* = 18)	Group 2 > 12 years (*n* = 8)	*P*-value
**Attention**			
Mean ± SD	15.8 ± 2.6	15.6 ± 2.1	0.885
**Memory**			
Mean ± SD	19.1 ± 3.2	16.9 ± 3.3	0.123
**Fluency**			
Mean ± SD	6.8 ± 3.9	5.9 ± 2.3	0.555
**Language**			
Mean ± SD	22.4 ± 3.5	23.5 ± 2.4	0.531
**Visuospatial**			
Mean ± SD	13.4 ± 2.7	13.4 ± 2.4	0.950
**Total**			
Mean ± SD	77.5 ± 9.7	75.3 ± 9.5	0.550

*SD* standard deviation, *n* number

## Discussion

This study was designed to detect cognitive dysfunction in IGE patients, and its correlation with brain parenchymal sonography as an indicator for brain atrophy.

Idiopathic generalized epilepsies are a relatively common type of epilepsy in children and adolescents [2]. However, cognitive impairment is related to longer disease duration and higher frequency of seizure [11]. The age group included in this study was selected for early detection of cognitive impairment in IGE patients. The results showed that there was a significant lower score in attention, memory, verbal fluency, and total scores of ACE-III in cases in comparison to the controls. However, no significant differences were detected between patients and controls in language and visuospatial scores. Javurkova and his colleagues reported different domains of cognitive deficits in IGE syndromes [12].

The study results showed that TCS findings, including parameters of brain atrophy (diameter of third ventricle, diameters of right and left anterior horn of lateral ventricle), showed no significant correlation with attention, memory, fluency, language, visuospatial or total score of ACE-III in the patient group. On the contrary, Seidenberg et al. noticed that cognition showed significant association to parenchymal structures in unilateral TLE [13]. This discrepancy may be attributed to smaller sample size in our study and the early stage of cognitive impairment in the included patients. Helmstaedter and Witt reported that the impact of epilepsy on cognition should be considered in relation to the underlying brain pathology and its dynamics, rather than solely the duration of epilepsy [14]. This perspective supports our findings, as the duration of epilepsy showed no significant correlation with cognitive domains measured by ACE-III.

The current study also noticed that seizure frequency was significantly correlated with cognitive assessment in the epileptic patients, consistent with previous studies suggesting that frequent seizures are related to the dysfunction in memory and executive function domains [15–17]. Frequent seizures can lead to hypoxia of the neuronal cells and brain dysfunction, resulting in cognitive function decline [17].

Our study showed that the third ventricle diameter is significantly larger in patients with epilepsy compared to healthy controls (*P* = 0.004). Also, the diameters of right and left anterior horn of lateral ventricles were significantly increased in epileptic patients compared to control group. The lateral walls of the third ventricle are formed by the thalami bilaterally, suggesting that alterations in third ventricle diameter might reflect the structural changes in the thalami. The thalamus plays a role in different cognitive domains [18], indicating that TCS might be a useful method for the assessment of ventricular diameter and its enlargement serving as a marker for brain atrophy and cognitive impairment.

However, this study has limitations, including a small sample size, the use of limited types of cognitive tests, and investigating certain epilepsy types. For future research, we would recommend a larger cohort, a broader range of cognitive tests, and the inclusion of different epilepsy types to further validate these findings.

## Conclusions

This study concludes that IGE patients exhibit cognitive dysfunction regarding attention, verbal fluency and memory, which is associated with brain atrophy compared to healthy subjects. Additionally, the frequency of seizures is significantly correlated with cognition in IGE patients.

## Data Availability

The datasets generated and/or analyzed during the current study are not publicly available due to the current Cairo University regulations and Egyptian legislation, but they are available by a reasonable request from the corresponding author.

## Abbreviations

ACE-III: Addenbrooke's Cognitive Examination-III
CSF: Cerebrospinal fluid
EEG: Electroencephalogram
EGTCS: Generalized tonic colonic seizures only
IGEs: Idiopathic generalized epilepsies
GGEs: Genetic generalized epilepsies
GTCs: Generalized tonic–clonic seizures
JME: Juvenile myoclonic epilepsy
MRI: Magnetic resonance imaging
SD: Standard deviation
SPSS: Statistical Package of Social Sciences
TCS: Transcranial B-mode sonography
